# *Mycobacterium avium* Complex Infection Imitating Whipple Disease in an Immunocompromised Patient With Newly Diagnosed Acquired Immunodeficiency Syndrome

**DOI:** 10.14309/crj.0000000000000588

**Published:** 2021-05-12

**Authors:** Subin Chirayath, Hammad Bin Liaquat, Janak Bahirwani, Atef Labeeb, Kimberly Chaput, Chatargy Kaza

**Affiliations:** 1 Internal Medicine Department, St. Luke's University Hospital Health Network, Bethlehem, PA; 2 Gastroenterology Department, St. Luke's University Hospital Health Network, Bethlehem, PA; 3 Pathology Department, St. Luke's University Hospital Health Network, Bethlehem, PA

## CASE REPORT

**Figure 1.**
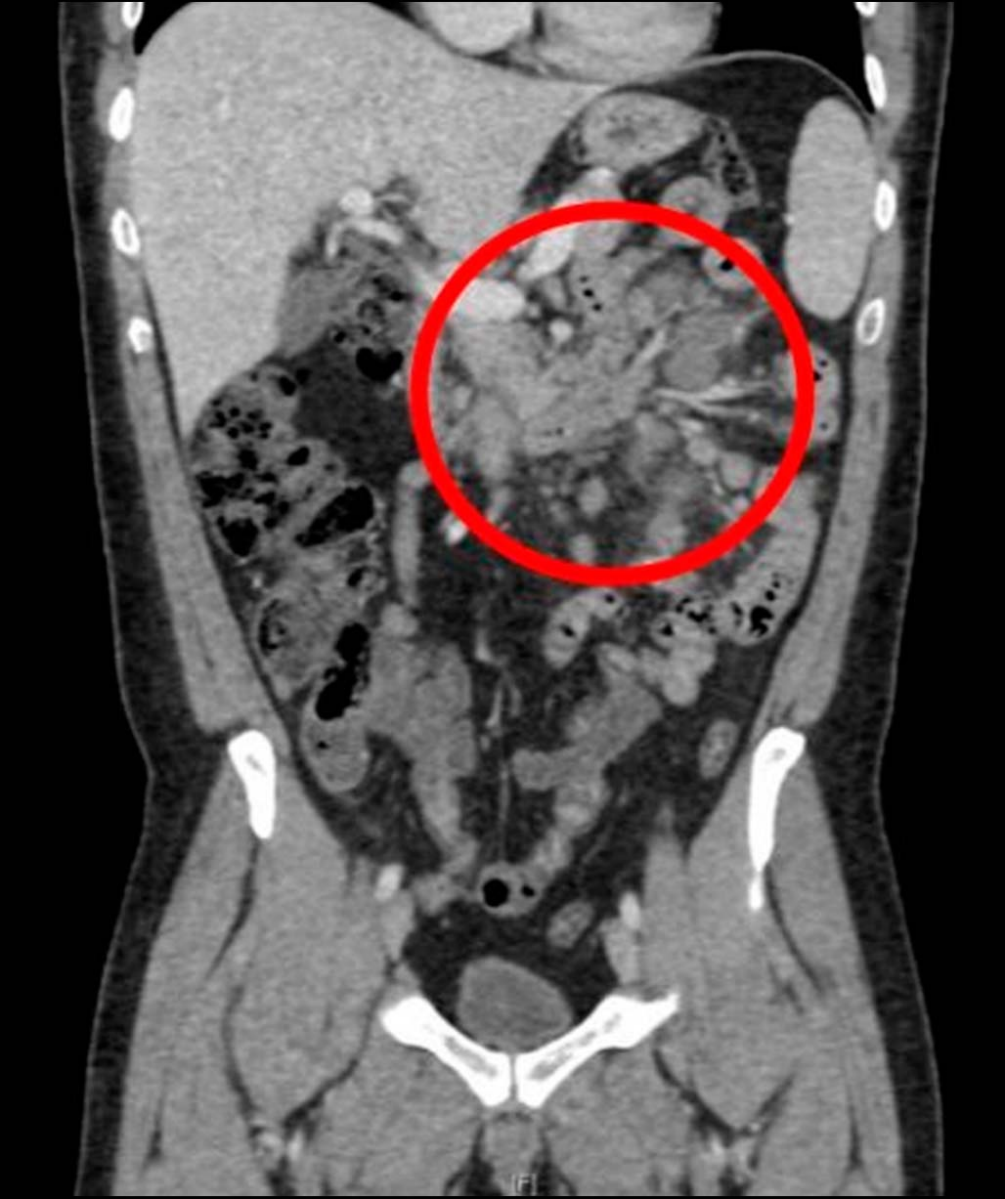
Abdominal lymphadenopathy noted on abdominal computed tomography, as indicated by red circle.

**Figure 2.**
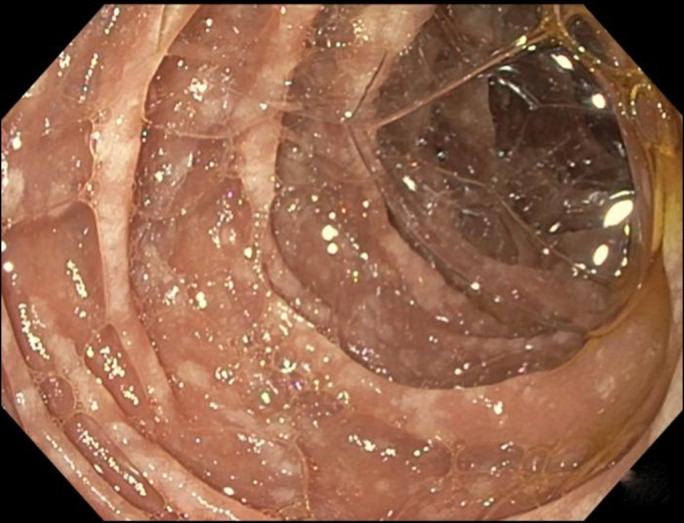
View of duodenum during endoscopy which shows white color plaques in the second and third part of the duodenum.

**Figure 3.**
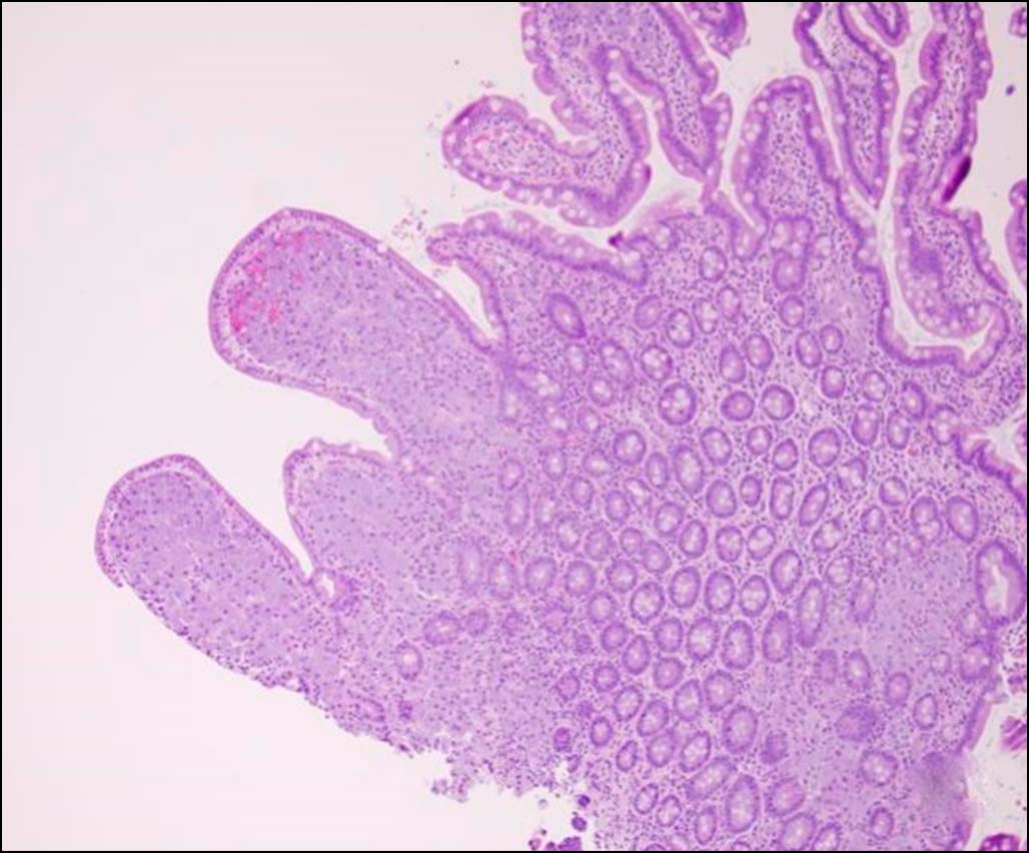
Hematoxylin and eosin stain shows mildly blunted villi because of expansion of lamina propria by foamy macrophages in a patchy fashion, 20×.

A 37-year-old homosexual man presented with a 1-year history of progressing abdominal pain and a 60-pound unintentional weight loss, reporting that he was in a monogamous relationship with 1 male partner. Initial complete blood count showed leukopenia at 3.58 K/μL and anemia at 10.6 g/dL, whereas the comprehensive metabolic panel showed decreased albumin at 2.5 g/dL and hypokalemia at 3.3 mmol/L. Abdominal computed tomography showed retroperitoneal and mesenteric lymphadenopathy (Figure 1). An esophagogastroduodenoscopy revealed abnormal appearing duodenal mucosa with duodenal biopsy revealing blunted villi and infiltration of foamy macrophages with periodic acid-Schiff positivity, suggestive of Whipple disease (WD) (Figures 2 and 3). Because of the characteristic appearance of the biopsy and his clinical symptoms, a presumed diagnosis of WD was made, and he started on intravenous ceftriaxone.

**Figure 4.**
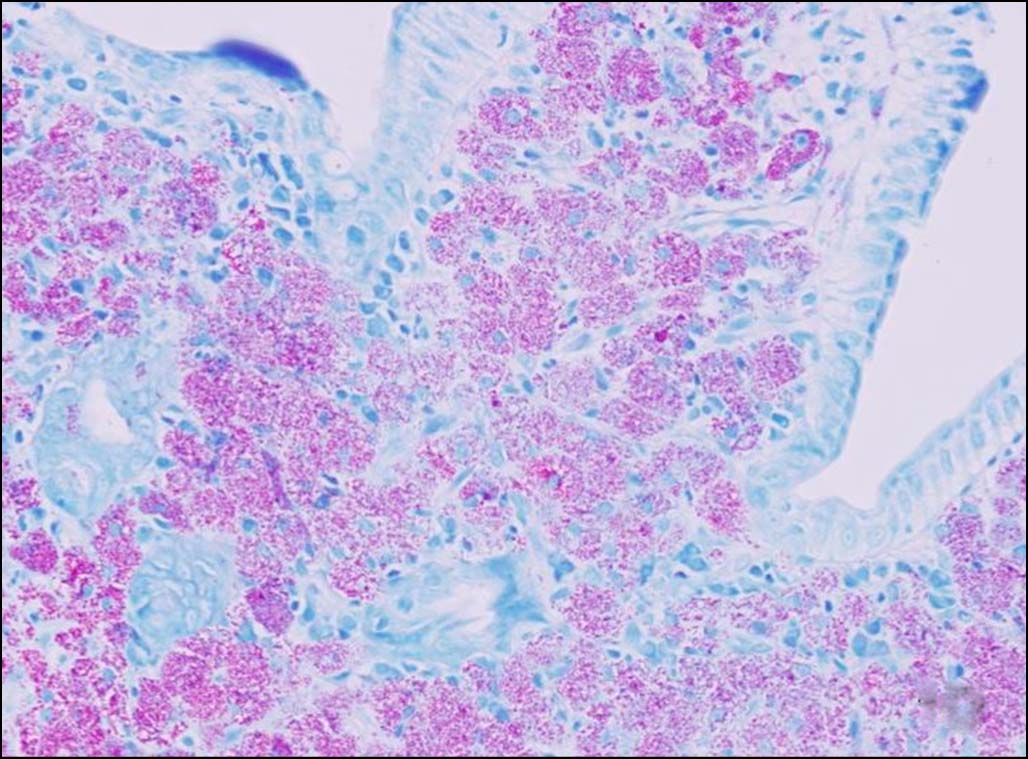
Acid-fast bacilli stain shows many “positive bacillary forms” confirming the presence of *Mycobacterium avium* intracellular complex, 20×.

The patient was discharged on oral trimethoprim/sulfamethoxazole but represented to the hospital shortly afterward with worsening symptoms. A human immunodeficiency virus test, drawn during his initial admission resulted after discharge and revealed a CD4 count of 9 and a viral load of >100,000 copies/mL. In light of this information, his duodenal biopsy was reviewed again and was found to be acid-fast bacilli positive consistent with *Mycobacterium avium* complex (MAC) enteritis (Figure 4). Blood cultures drawn on his second admission grew MAC supporting the diagnosis of disseminated disease. Appropriate treatment with azithromycin, rifabutin, and ethambutol and antiretroviral therapy were initiated, resulting in clinical improvement.

Patients with acquired immunodeficiency syndrome are susceptible to numerous opportunistic infections, including MAC, particularly with patients with CD4 counts less than 50.^1^ Usually presenting as a disseminated disease with fever, night sweats, diarrhea, and lymphadenopathy, it can mimic various other diseases, such as WD. WD is caused by *Tropheryma whipplei* and causes abdominal and joint pain, and a malabsorptive diarrhea.^2^ Because of the relative rarity, a diagnosis of MAC infection may be delayed, especially when presenting symptoms are nonspecific. MAC infection can present as localized or disseminated. Disseminated disease is more common and increases mortality in patients with acquired immunodeficiency syndrome threefold.^3^ By contrast, patients with WD have additional unique symptomatology, such as polyarticular arthritis which is present in up to 67% of cases. Patients with the 4 chief symptoms of diarrhea, weight loss, fever, and arthralgias should undergo evaluation.^4^ Periodic acid-Schiff stains can be positive in MAC infections because of macrophage-containing mycobacteria through phagocytosis leading to a misdiagnosis.^5^ Despite the decline in incidence, MAC infection should still be considered in immunocompromised patients presenting with vague generalized symptoms, weight loss, and lymphadenopathy.

## DISCLOSURES

Author contributions: S. Chirayath wrote the article. H. Bin Liaquat, J. Bahirwani, K. Chaput, and C. Kaza edited the article. A. Labeeb provided the pathology images. H. Bin Liaquat is the article guarantor.

Financial disclosure: None to report.

Informed consent was obtained for this case report.
